# A Multiphase Implementation of Ring Treatment: A Community-Engaged Control Program for T. Solium taeniasis/cysticercosis in Northern Peru

**DOI:** 10.21203/rs.3.rs-10132093/v1

**Published:** 2026-09-16

**Authors:** Lisset Dumet Poma, Percy Vilchez, Angela Spencer, Ruth Atto, Vanessa Cruz, Hector Garcia, Javier Bustos, Gracelyn Cruden, Seth O’Neal

**Affiliations:** Chestnut Health Systems; Universidad Peruana Cayetano Heredia; University of Washington; Universidad Peruana Cayetano Heredia; Universidad Peruana Cayetano Heredia; Universidad Peruana Cayetano Heredia; Universidad Peruana Cayetano Heredia; Chestnut Health Systems; Oregon Health & Science University – Portland State University

**Keywords:** Rural settings, low-income, infectious diseases, community-engaged, multisectoral collaboration, public health intervention

## Abstract

**Background:**

Cysticercosis (CC) is a zoonotic disease transmitted between pigs and humans, caused by the larval stage of T. solium that leads to neurological morbidity in humans and economic losses in pig-farming communities in low-resource rural regions. Ring Treatment (RT) is an evidence-based intervention that integrates a community participatory approach to provide antiparasitic treatment to households within 100-meters of an infected pig. This study aimed to evaluate the multiphase implementation of RT as a public health program in northern Peru, with a focus on feasibility, adoption, and sustainability.

**Methods:**

We conducted a multi-method, three-phase implementation study from 2019–2025 in Tumbes, Peru. Phase 1 involved a formative evaluation using the expanded Consolidated Framework for Implementation Research for low- and middle-income countries. We used semi-structured interviews (n = 169), social network surveys (n = 341), and two process-mapping workshops (n = 26) to describe barriers, facilitators, and RT protocol adaptations. In phase 2, we piloted RT in a small health district, reviewed registries, and conducted community surveys and interviews for quality improvement. In phase 3, we rolled out RT in two districts and applied the RE-AIM framework to assess implementation outcomes. Quantitative data were analyzed using descriptive statistics, while qualitative data underwent rapid qualitative analysis and template analysis (thematic analysis for hierarchical coding).

**Results:**

Key facilitators included adaptability of existing health programs and strong engagement with community health workers (CHW) and communities. Barriers included high staff turnover, workload, and resource constraints. The pilot study achieved 75% treatment coverage among eligible populations, increased community knowledge of the CC transmission cycle, and prompted regional-level policy changes. During the RT rollout, 100% of health facilities established RT procedures, with moderate participation in training and treatment (53% and 87%). However, the COVID-19 pandemic and political instability limited the effectiveness of assessments and the completeness of data.

**Conclusions:**

RT is feasible and acceptable as a public health program for *T. solium* control in rural, endemic, low-resource settings. Success hinges on community and CHW involvement, adaptive protocols, and cross-sectoral partnerships. Continued investment in One Health approaches that view the human-animal-environment intersection as central to health is needed to sustain and expand progress in controlling zoonoses.

## INTRODUCTION

Zoonoses, infectious diseases transmitted between animals and people, cause devastating health and economic effects in low-resource settings. ^1, 2^ Cysticercosis (CC) is a zoonotic disease transmitted between pigs and humans caused by the *Taenia solium* (*T. solium*) parasite, also known as pork tapeworm. ^3^ Neurocysticercosis (NCC), a central nervous system *T. solium* infection, can cause headache, epileptic seizures, and intracranial hypertension. In South American and African low-income rural areas, NCC is responsible for 30% to 50% of acquired epilepsy.^4–6^ NCC also imposes substantial costs on human productivity by lost income and unemployment, and pig infections (porcine cysticercosis) are a food safety hazard and source of economic losses in rural farming areas.^7, 8^ CC is more prevalent in rural low-income communities with underdeveloped sanitation infrastructure, open defecation practices, and no livestock corrals.^9^ Often, these communities also lack healthcare infrastructure and financial stability to control disease.^1^

A variety of population-level strategies have been tested to control or prevent taeniasis (human intestinal infection with adult-stage *T. solium* and the direct cause of both NCC and porcine CC). These include mass drug administration (MDA) to humans and/or pigs,^10–13^ mass or targeted screening and treatment,^13–16^ pig vaccination,^17^ education, and improved sanitation.^18–20^ Interventions to control or prevent *T. solium* transmission require comprehensive approaches that account for the interrelationships among human, animal, and environmental risk factors, as well as the socio-political and economic contexts of rural low-income areas in which these risk factors arise.

**Figure 1:**
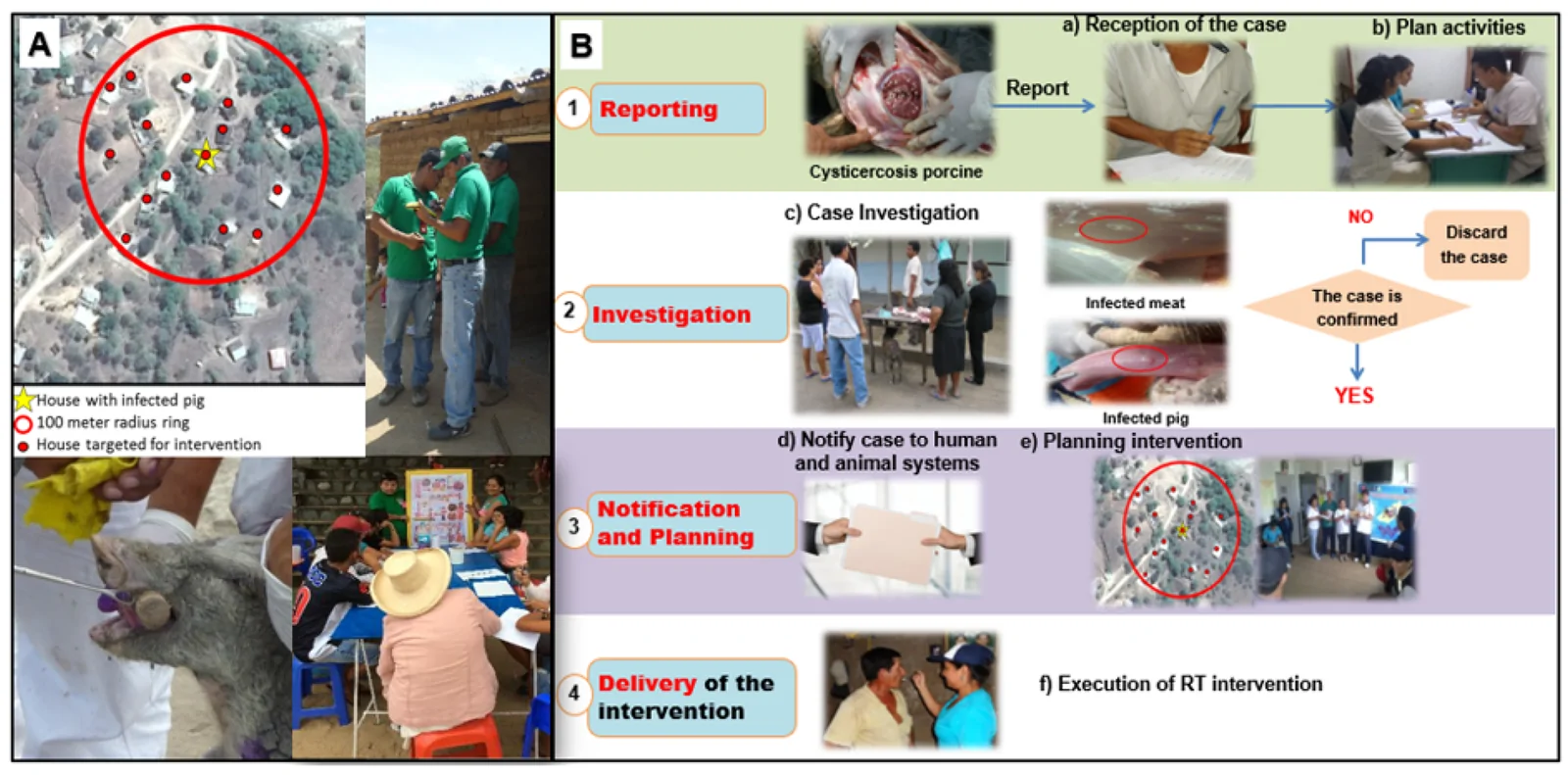
A: The Ring Treatment (RT) intervention; B: RT flowchart with main health system activities

Over decades of collaborative research activities, the Cysticercosis Working Group in Peru,^21^ developed and assessed the RT intervention to control CC. RT integrates a community approach to surveillance for pig and pork infections and subsequent antiparasitic drug delivery, and accounts for the context of rural low-income settings. RT leverages the strong spatial clustering of CC between human and pig hosts.^22^ Taeniasis prevalence is estimated to be at least eight times higher among people living within a 100-meter radius of a heavily-infected pig with CC than among people living outside that radius.^23^ Additionally, using GPS tracking devices in pigs, a study found that 100-meter-radius rings adequately capture the average pig’s roaming area and represent the area where the great majority of exposure to human feces occurs.^24^ In RT, a safe and efficacious antiparasitic drug, niclosamide (NSM),^25^ is delivered to a population subgroup at higher risk of taeniasis: household clusters near heavily-infected pigs, identified by visual inspection of pork at slaughter or tongue examination of live pigs (Fig. 1, section A). RT’s evidence base is strong — it can reduce *T. solium* transmission by ~ 50% within one year, is comparably effective to MDA with antiparasitic treatment for the entire community, yet requires only a small fraction of drug doses.^14, 22^

RT’s core health system activities are organized into four steps (Fig. 1, section B). The first step is receiving a report of an infected pig or pork. Step two is case investigation and confirmation by a trained health worker, community veterinarian, or environmental health specialist. In resource-poor areas, heavily-infected pigs are often identified at slaughter, and live pigs can be inspected for cysts by examining the tongue for visible or palpable nodules.^26, 27^ Residents who identify pig infections are encouraged to report the case in person, by phone, or by text to a community health representative or the nearest health care facility. The infection is confirmed or ruled out by checking cysts in the meat or the pig’s tongue. If confirmed, step three is to notify animals’ systems or the local veterinarian of the case, and determine the ring (i.e., radius of treatment) of households to offer NSM. Health care providers and community health workers (CHWs) collaborate to plan how to reach the households located in the treatment ring. Step four entails administering NSM, providing hygiene-practice recommendations, and conducting follow-up visits to monitor for adverse events. Step four may require several visits to the participating households until the target treatment coverage is reached. Based on previous mathematical studies, coverage is achieved when 70–80% of the eligible population receives at least one dose of NSM.^28, 29^

Two main processes must occur for RT implementation to reduce parasite transmission. First, there must be a functional surveillance system to detect heavily-infected pigs. Without case detection, no intervention rings are formed, and NSM is not delivered to the community. Second, there must be an efficient mechanism for local health facilities to deliver NSM to at-risk people residing in the ring areas. In previous studies that evaluated the feasibility and effectiveness of RT as a community-engaged surveillance system, we demonstrated that 1) community residents can fulfill the role of surveillance and reporting if provided adequate education and incentives, and 2) local health facilities can deliver RT if provided with defined protocols and adequate support.^30–32^ We also identified three core activities to facilitate a CC community surveillance: (1) Participatory education workshops based on local epidemiologic and physical evidence, (2) Providing incentives to villagers to report infected pigs, and (3) Identification and training of local champions, such as CHW, to promote surveillance and receive reports. These core activities informed the implementation strategies selected for future research. The study reported here aimed to adapt the processes and protocols established in the research setting to the programmatic context with input from key stakeholders.

### Implementation of Ring Treatment (RT) in Peru

**Figure 2:**
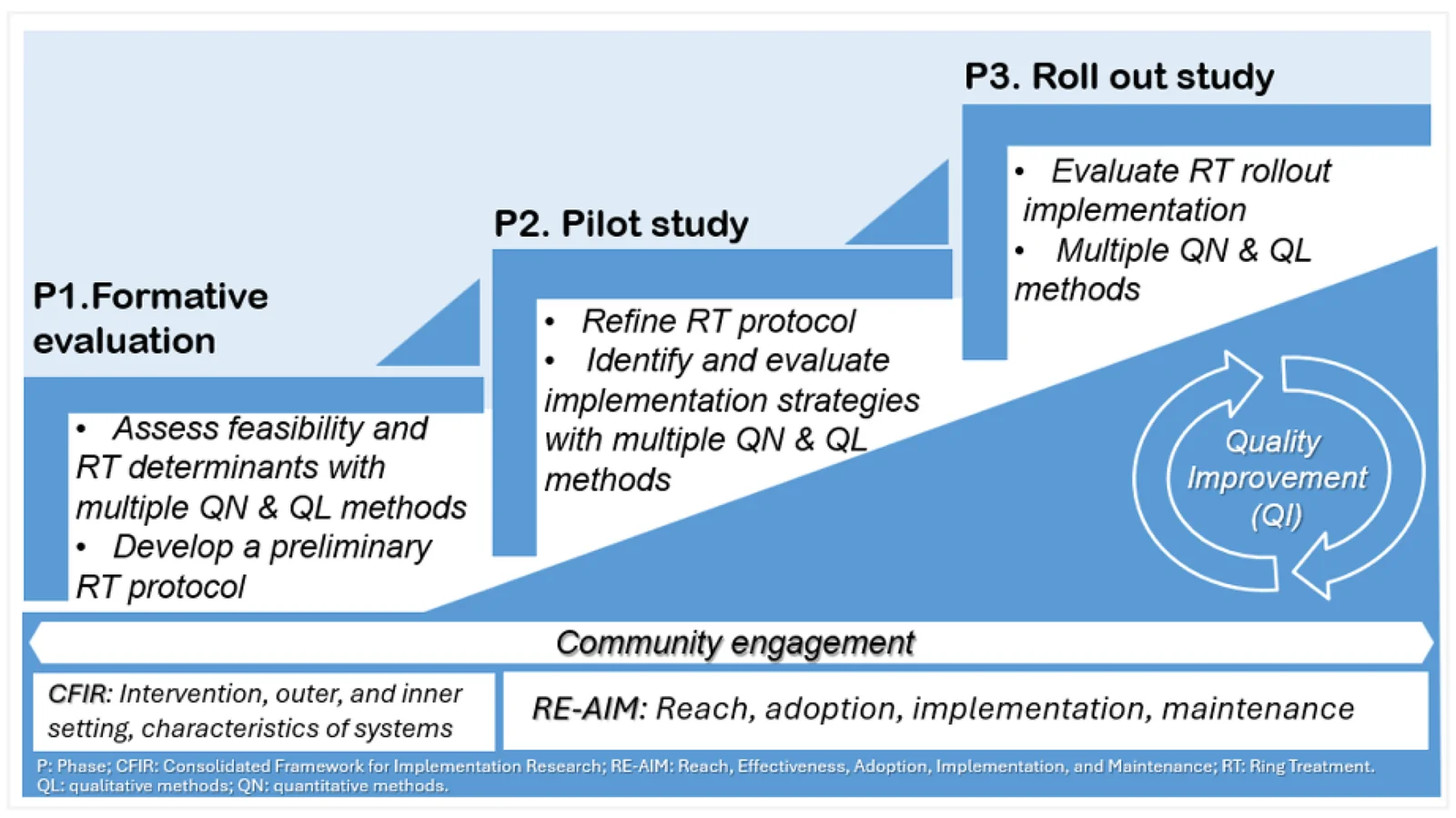
Overview of the multiphase RT implementation in northern Peru

The prevalence of CC in rural areas of northern Peru is high, with circulating antibodies suggesting prior exposure in up to 36.9% of individuals in typical endemic settings.^4^ Small-population studies using computerized tomography scans in adults in rural endemic towns have documented that 10-18.8% have evidence of current or prior brain infection.^4, 33^ While the previously described project demonstrated RT’s effectiveness in controlling *T. solium* taeniasis/cysticercosis when delivered by research staff, its effectiveness when offered as a governmental health program was untested. To address this gap, a 5-year project funded by the US National Institutes of Health (2R01NS080645) was conducted in northern Peru (Fig. 2) from 2019 to 2025 (extended due to the COVID-19 pandemic). To implement RT in multiple stages, we applied principles of quality improvement and community engagement to select and assess implementation strategies.^30–32, 34^ First, we used the Consolidated Framework for Implementation Research (CFIR), extended for Low- and Middle-Income Countries (LMICs), to conduct a formative evaluation of RT feasibility and to assess health systems’ capacity.^35, 36^ Subsequently, we conducted a pilot study in a small health district to iteratively evaluate activities and develop and refine an RT protocol.^34^ Finally, we rollout RT in three districts and evaluated implementation using the RE-AIM framework (reach, effectiveness, adoption, implementation, and maintenance).^37^ All three phases were conducted in Tumbes, Peru. Ethical approval was obtained from Oregon Health and Science University (IRB ID: STUDY00024870) and Cayetano Heredia University (IRB ID: 200738).

This paper aims to summarize the methods and main outcomes of the RT formative evaluation (phase 1), the RT pilot study (phase 2), and the RT rollout across three districts (phase 3). We organized this paper into three sections, providing an overview of the methods and the corresponding results in each phase.

## PHASE 1: FORMATIVE EVALUATION OF RT IMPLEMENTATION

### Formative Evaluation Methods

Our formative evaluation aimed to assess the feasibility of RT implementation in Tumbes, Peru’s public health system. We applied multiple quantitative and qualitative methods, including: 1) a template analysis (thematic analysis for hierarchically coding) of semi-structured interviews to describe the barriers and facilitators of RT implementation^38^ informed by a version of the CFIR with added constructs relevant in LMIC contexts;^35^ 2) social network analysis to identify key players for RT adoption; and 3) process mapping to develop RT delivery protocols in alignment with organizational contexts. Table 1 summarizes the methods used, and further details are in Additional Document A.1. The formative evaluation was conducted among four health centers and eleven health posts that are part of a rural healthcare delivery network serving small, medium, and large municipal districts in Tumbes, Peru (population served by district size: <5000, < 6000, and < 36000, respectively). Recruitment occurred through visits to health facilities and meetings with governmental and community leaders from human, animal, and environmental systems from January 2020 to January 2022. These leaders consented to participate in semi-structured interviews.

Semi-structured interviews were conducted in Spanish, audio-recorded, and transcribed. They lasted approximately one hour, and collected information across the CFIR, including intervention constructs (e.g., relative advantage, adaptability), inner setting (e.g., tension for change compatibility), and extended constructs related to system characteristics (e.g., resource continuity, community characteristics). Five analysts, including three site interviewers, analyzed the transcripts using template analysis and identified quotes representing relevant CFIR constructs using Atlast.TI software. Each team member assigned the strength and valence of each construct (from − 2: strong negative influence on implementation; 0: neutral influence; 2: strong positive influence), and we obtained an average per construct to identify barriers and facilitators to RT implementation.^39^

Social network surveys collected nominations for which person, unit, or organization participants deemed best suited to carry out each RT step. We assessed the direction of nominations using network centrality measures, such as in-degree (i.e., the number of nominations received by an individual), to identify the key actors considered best to perform the implementation task. Hence, those with the highest scores had more peers who considered them the best at performing the task. We used RStudio 4.4.2 for data transformation and UCINET 6 to obtain SNA measures and network figures.^40^

Process mapping is one of the key elements of the Systems Analysis and Improvement Approach, an innovative systems engineering strategy for improving the implementation of evidence-based interventions across different settings.^41^ It facilitates understanding of practices and processes into which new interventions are introduced to advance patient outcomes and improve health care efficiency.^42^ To conduct process mapping, we facilitated a workshop with healthcare staff, including the health center’s chiefs, CHWs, and others involved in RT implementation. Participants discussed RT processes and how to adapt them to their programs, who should oversee each step to validate SNA results, and possible reporting tools. The research team reviewed the diagrams developed by participants, including proposed changes, condensed them into a single diagram in Microsoft PowerPoint, and subsequently shared it with participants.

### Formative Evaluation Results

**Figure 3:**
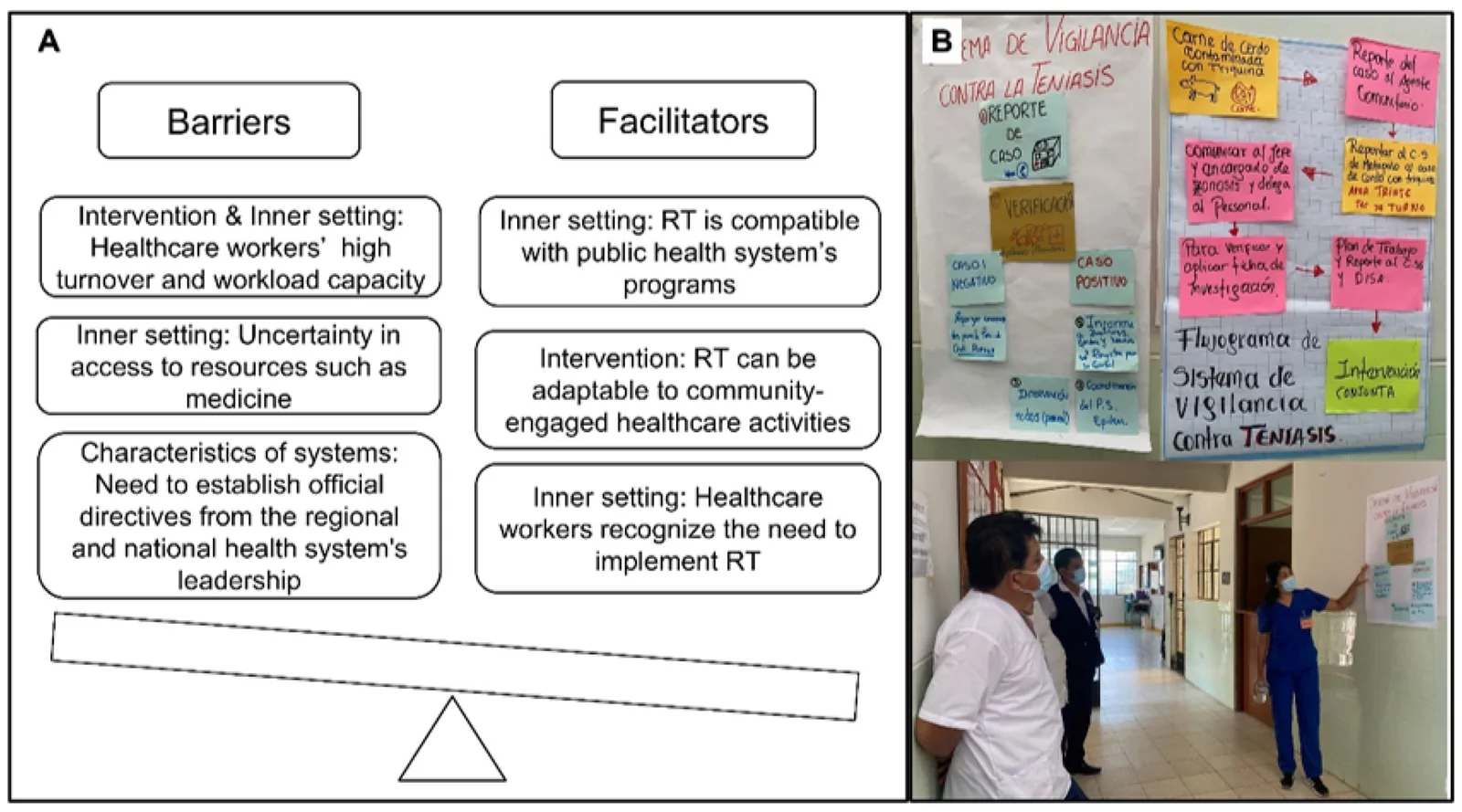
A. Results of barriers and facilitators of RT implementation - CFIR analysis. B. Pictures from a process mapping workshop

Figure 3 summarizes the barriers and facilitators of RT implementation. Facilitators included RT adaptability, health system learning climate, and compatibility. Participants reported that RT is compatible with current public community-engaged health care programs and system processes. We also found that participants recognized the burden of zoonoses, including CC, and their impact on communities and animals, underscoring the need to apply One Health approaches that view the human-animal-environment interface as central to health. Factors that hindered implementation were within the inner setting, including workload capacity, costs, available resources, and high health care worker turnover. Participants also described constructs in the LMIC-extended CFIR related to system characteristics, such as authority norms—the need to establish official directives from the regional and national health system leadership to support adaptations of surveillance systems and to formalize partnerships between human and animal public health systems. A summary of the CFIR construct’s strengths and valences is in Additional Document A.2.

Social network analysis allowed us to map connections among health and animal systems and across these systems with the community, and to describe the roles and functions that should oversee each RT step. We identified CHWs as the most frequently nominated to receive reports of infected pigs or meat with cysticercosis from the community, thus serving as the link between the community and health care systems. We also identified key players who were nominated as best at incentivizing case reporting, such as the mayor and local government workers in charge of environmental health or sanitation. Process mapping provided more elaboration on possible roles and units responsible for each RT activity beyond what was described in the SNA, as well as current systems, tools, or forms for capturing activity outputs. Adaptations in human and animal surveillance systems were also identified, including additional health systems formularies to capture suspected cases of porcine cysticercosis and investigation results; a preventive treatment administration log for taeniasis; an NSM epidemiological form; and a monthly consolidated report of porcine cysticercosis cases. Formative evaluation results were shared with health care decision-makers through reports and meetings, and informed implementation strategies in subsequent phase activities, further described below, such as participatory workshops. Additional Document A.2 includes more detailed figures and tables of the SNA and process mapping results.

A preliminary RT delivery protocol was developed using a national regulatory guidelines framework, supported by charts co-developed through process mapping, combined with SNA and themes on barriers and facilitators from interview analysis. The protocol provides guidelines for RT delivery, identifies the health systems units that should lead each RT delivery process, and outlines the changes required in surveillance systems to capture RT activities and outcomes.

## PHASE 2: PILOT STUDY OF RT IMPLEMENTATION

### RT Pilot Study Methods

Two main components must be established for successful RT implementation. First, there must be a functional community surveillance system to detect heavily infected pigs. Without case detection, no intervention rings form, and NSM is not delivered to the community. Second, local health facilities need established processes and well-trained personnel to deliver NSM to individuals residing in the ring areas. Thus, the main goal of the RT pilot study was to identify and evaluate strategies to facilitate the establishment of community-engaged surveillance systems and case-reporting incentives, and to adapt RT processes to the local programmatic setting, including NSM treatment-delivery planning and execution. We also aimed to refine the RT implementation protocol to inform regional *T. solium* taeniasis/cysticercosis control guidelines.

The pilot study was conducted from February 2022 to February 2023 in a single small health district, serving 5,279 people across seven villages. We selected this district because its limited access to health care resources and infrastructure enabled us to capture challenges that may not be encountered in larger districts with fewer financial constraints. We used quality improvement methods informed by the RE-AIM framework to evaluate and iteratively improve the working RT implementation protocol on a 3-month cycle.^34, 41, 43^ In Table 2, we expand on how we operationalized RE-AIM by mechanisms, methods, and data collection tools aligned with each implementation outcome and strategy. For instance, Reach was evaluated based on participation outcomes and attendance rates; thus, initial activities included introductory workshops on CC and RT for the selected district, providing guidance to increase motivation to participate among community members and health care workers. The research team assisted in delivering participatory workshops to community members, CHWs, and health systems providers. These activities aimed to increase community participation and were evaluated using attendance registries.

#### Fidelity

We used procedural checklists to monitor *adherence to the protocol* and key RT processes (e.g., case confirmation, carcass disposal, incentive provision, and determination of treatment-eligible households). Health workers completed checklists after each RT intervention. Summary statistics on RT delivery included the total number of participating health system staff (including CHWs) and community members with knowledge of case identification and the reporting system. Pig Infection registries were also completed to capture case report information, such as the location of the suspected infected pig or pork, and the carcass’s disposition. Health workers completed registries on an ongoing basis (e.g., weekly) to document actions taken and the timeliness of responses to case reports. Registry data were included in summary reports provided to health systems upon completion of each RT intervention for confirmed cases, thereby enabling their capture in the surveillance system. We set an 80% threshold (20/24 checklist items) to evaluate RT protocol fidelity.

#### Community knowledge, attitudes, and practices

We conducted household surveys at two points, selecting a random sample of 10% of household heads in villages receiving health facility services to evaluate knowledge, attitudes, and practices regarding community surveillance and reporting of pig infections, reflecting RT *adoption and reach*. The baseline survey took place in June 2022 (n = 117), and the follow-up in November 2022 (n = 143). We calculated descriptive statistics, assessed community support for the RT implementation activities, and identified recommendations for strategies to promote the reporting of suspected infections.

#### Internal and external factors influencing RT

We conducted semi-structured interviews with CHWs, health care workers, and regional authorities to gather insights into internal and external factors influencing RT implementation *effectiveness* and solicit suggestions for process improvement. Some of the recommended adaptations included optimizing staff availability through community activities to increase the likelihood that residents would be home. We also interviewed community residents to gather information about their experiences with surveillance and reporting of infected pigs, and their acceptance of NSD. We used purposive sampling to include three individuals who did not report infected pigs and three who did not accept treatment. Interviews were recorded and transcribed in Spanish. The research team reviewed transcripts and conducted a rapid qualitative analysis, resulting in a short report with recommendations for improvement that was shared with health system leaders.^44^

### RT Pilot Study Results

The research team held eleven educational workshops on the *T. solium* taeniasis/cysticercosis transmission cycle and the RT implementation process. Attendees included municipal and health authorities (n = 58). Eight community-engaged necropsy workshops were attended by 215 community residents. CHWs also received training on the disease transmission cycle and the RT surveillance system from health promotion workers at the local public health department (n = 62). Afterward, CHWs actively recruited 18 community members to be trained in the porcine tongue examination. The research team remained available throughout this phase to assist the health system in improving community knowledge of the disease and the surveillance system. Educational materials were also co-developed with guidance from the research team, including videos and audio that were broadcast to the community via radio and social media.

Community surveys showed increases in community knowledge of pig inspection (from 37% in the baseline assessment to 52% in follow-up) and in the case reporting process (from 28% to 44%). However, we identified gaps in knowledge about the *T. solium* parasite transmission cycle and its relationship to CC, as well as where to report cases. Results also identified recommendations to encourage case reporting, such as providing pig cost replacement or food baskets. Themes derived from rapid analysis of the semi-structured interviews signaled strong support for RT among residents and health staff, particularly among households that received the anti-parasitic drug NSM following a reported case. Interviews with local health care leaders and the mayor were consistent with survey results and helped identify implementation strategies to adapt community incentives to improve case reporting.

Five infected pigs were reported during this pilot study, prompting government-led NSM treatment within 5 100-meter treatment rings. RT coverage resulted in 75% (440/589) of the eligible population receiving treatment (Table 3).

Preliminary indicators for RT maintenance and sustainability from the pilot phase include support from the Ministry of Health for the regional rollout of RT. Demonstrating the strong relationship developed between the research team and the Ministry of Health, which served as a facilitator of future RT implementation efforts, the Ministry sponsored a workshop to disseminate community-engaged zoonoses control programs. Attendees included zoonoses leaders from the Ministry of Health at the local, regional, and national levels. The research team presented RT activities and preliminary outcomes at the workshop, along with an updated guideline for the provision of antiparasitic drugs for the control of taeniasis and cysticercosis. Informed by the protocol developed in the pilot study, the region of Tumbes enacted a technical norm that listed RT as an evidence-based intervention for controlling taeniasis and cysticercosis. Lastly, dissemination meetings were held with representatives of the Pan American Health Organization, which encouraged a national donation of NSM to the Peruvian Ministry of Health.

Based on the formative evaluation and pilot study, we identified a package of implementation strategies. These strategies include identifying early adopters, cultivating relationships with key leaders across human, animal, and environmental health sectors, and obtaining formal commitments from essential partners. The strategies also encompass community engagement through participatory meetings, training, and incentives, as well as structural adaptations to surveillance systems. We found it essential to develop regulatory documents, provide ongoing consultation, capture local knowledge, and foster collaboration through network weaving and top-down engagement with national and international health authorities, such as the Pan American Health Organization, to ensure a multisectoral, collaborative approach to implementation. See Additional Document A.3 for a comprehensive overview of the strategies, including definitions of the actors and specific actions.^45^

## PHASE 3: ROLLOUT OF RT AT THE DISTRICT LEVEL

### RT Rollout Methods

In the third phase, the rollout of RT, we assessed a regional rollout of the RT intervention using the RE-AIM framework to measure implementation outcomes in a shortened trial due to COVID-19-related delays (March 2023- August 2024). In this phase, we implemented RT in seven hospitals and 21 smaller health centers across two districts in Tumbes, Peru, considered endemic for taeniasis: District 1 (pop. 7000) and District 2 (pop. 5000). We used the same qualitative and quantitative methods described in the phase 2 pilot study — checklists, infected pig registry, surveys, and semi-structured interviews. However, data collection occurred annually and focused on assessing RE-AIM implementation outcomes. Reach was assessed through community and health system surveys. We collected 170 surveys across two-time frames (35 health care workers, 34 community health workers, and 101 community members) and calculated descriptive statistics.

To assess RT effectiveness, we planned to conduct a census of the pig population in the two selected districts and measure the sero-prevalence of antibodies against cysticercosis in a random, representative sample at two time points. Challenges posed by COVID-19 delayed data collection and prevented the second assessment from taking place. At the first point assessment, the team conducted a census of the population in the two selected districts: district 1 (4,596 people) and district 2 (6,821 people). Baseline serological sampling was performed on all pigs older than 2 months to identify T. solium cysticercosis, conducted from October 2023 to February 2024. A total of 1,470 pigs were sampled (district 1: 903 samples; district 2: 567 samples), and subsequently, 600 serological samples were randomly selected.

Lastly, we examined barriers and facilitators to RT implementation through seven semi-structured interviews with three community members, three health care workers, and one person responsible for animal epidemiological surveillance. Community interviewees included both participants and non-participants in RT and the NSM usual care treatment.

### RT Rollout Results

Table 4 summarizes results from surveys, registries, and checklists used to evaluate RT implementation. Reach was measured with participation rates; all seven health facilities participated in training and RT delivery. Some of the implementation strategies activities to support communication about the RT and improve knowledge of *T. solium* transmission cycle included seven introductory workshops on taeniasis/cysticercosis and RT intervention, and three participatory pig necropsy workshops, supported by research teams across the two health districts, resulting in 110 health professionals and 64 CHWs being trained. The research team also provided training to eight of the ten physicians from participating in health care facilities on human neurological assessment for CC, as requested by the health care director in one participating district. These activities were not directly related to RT activities but were intended to support relationship-building with health care facilities.

One case of CC-infected pork was reported during phase 3. Interviews with community members and health care workers indicated strong commitment and collaboration among health and animal systems personnel. Health care staff emphasized the feasibility and simplicity of the RT process and of compatibility with tasks like those in other community public health programs. Participants reported that other key community actors supported them in managing the reporting process, including the former lieutenant governor and a local butcher, while CHWs found case reporting manageable. All seven facilities established procedures for RT. The animal health coordinator and veterinarian played a vital role by conducting site visits and ensuring cases were documented and managed in accordance with established guidelines. Some barriers were also described in the RT implementation processes. The community noted insufficient dissemination of information about the RT surveillance system, resulting in gaps in awareness among community members. CHWs felt they needed continuous capacity-building. A shortage of personnel was reported during the intervention phase, potentially limiting the effectiveness and reach of RT and other zoonosis control activities. These barriers highlight areas for improvement in communication, training, and resource allocation to strengthen future surveillance efforts.

Effectiveness was not included in the final assessment due to substantial delays caused by the COVID-19 pandemic, which compromised our ability to conduct a second assessment of pig CC seroprevalence. In the baseline assessment, a random selection of 600 serological samples was processed to assess the sero-prevalence of antibodies against cysticercosis in pigs in the two selected districts, obtaining the following results: 474 pigs got a negative result for exposure to cysticercosis (0 bands) and 126 pigs got a positive outcome ( > = 1 bands; 1 band = 92 pigs; 2 bands = 20 pigs; and > 3 bands = 14 respectively). As indicators of RT implementation maintenance, two municipal ordinances were passed in the two participating districts. Ordinances regulated husbandry and hygiene practices, established a process for inspection of the slaughtering of domestic farm animals, as well as proposed sanctions for unreported infected pigs or meat.^46, 47^ Throughout the study, we provided a series of didactic and applied implementation science training for trainees and interested parties in human, animal, and environmental health to advance implementation research capacity and promote RT maintenance in Peru. Further, we supported four doctoral students and two master’s students, including those specializing in epidemiology. We also conducted three public workshops on implementation science in Peru.

## DISCUSSION

This paper describes the results of the RT multiphase implementation RT, an evidence-based intervention for controlling *T. Solium* taeniasis/cysticercosis. We summarized the methods and results from a formative evaluation, a pilot implementation study in a small district, and the rollout of RT in two districts in Tumbes, Peru. Despite the disruption caused by the COVID-19 pandemic, RT implementation outcomes are promising overall. Contextual conditions were essential to support this outcome, including the substantial scholarship and long-standing partnership developed by the Cysticercosis Working Group in the region.^21^ In Phase 1, the formative evaluation, we identified key players to support RT implementation, collected essential information for the development of RT protocols, and identified potential implementation strategies to be iteratively refined in Phase 2. Through qualitative analysis of interviews, we identified barriers and facilitators to RT implementation, including RT’s compatibility with public health programs that use community-participation approaches, as well as inner setting barriers such as high workload and turnover. Implementation studies in LMIC contexts have reported similar findings regarding inner-setting barriers and organizational factors.^48–50^ These limitations were exacerbated by the COVID-19 pandemic and the country’s political turmoil — during our project timeline (2019–2025), Peru had five different presidents and 13 ministers of health, leading to high turnover among regional government health directors and delays in public services due to policy paralysis.^51^

Our study used the extended CFIR framework for LMICs (34), which helped identify barriers related to hierarchical organization, authority norms, and siloed systems. For example, we observed conflicting rules between the municipal and livestock agencies for verifying the status of infected pigs/meat, a lack of collaboration with environmental and sanitation agencies, and differing levels of engagement among human health, livestock, and municipal leaders. As a result, local studies were conducted in the Peruvian setting to examine policy processes across the systems involved in zoonoses management, providing essential information to inform strategies to reduce system silos and facilitate RT implementation.^52, 53^ A multiple-methods study involving human, animal, and environmental policy makers found that a nationwide policy establishing the One Health approach, and an integrated approach that aims to sustainably balance and optimize the health of people, animals, and ecosystems, was needed to address zoonoses and guide multisectoral work.^52, 54^ Furthermore, results from those studies recommended local regulations as accessible tools for institutionalizing roles in zoonoses multisectoral activities.^53^ Thus, implementation strategies in our project focused on providing consultation on the development of regulatory documents, directives, or ordinances for local and regional policymakers, especially to guide agricultural sanitary behaviors and incentivize CC pork case reporting.

Our team also followed a top-down approach as an implementation strategy, including promoting network weaving and engaging and disseminating study results with regional, national, and international leaders, which helped alleviate study challenges. Some of the collaborative work included meetings with the Pan American Health Organization, the Ministry of Health, and the Tumbes Regional Health Directorate to develop a technical dossier to justify the NSM donation. Efforts ensured access to medication during the implementation of RT in study villages, as well as support for other national zoonoses public health programs in neighboring regions, such as Piura. It also incentivized policy changes, such as the inclusion of NSM in public programs to control CC in Peru. The partnership and trust developed throughout the study in the region can open opportunities for future research to evaluate an RT implementation strategy with the One Health approach, which emphasizes cross-sectoral collaboration among human, environmental, and animal systems, in other endemic areas.^54^

Results from the phase 2 RT pilot study and phase 3 RT rollout highlighted the essential role of community key players, such as CHWs, in establishing trusting relationships with the community, disseminating public health information, serving as a link between the community and the health system, supporting implementation activities, and incentivizing case reporting. Similar implementation studies have identified the importance of CHWs freeing up clinic staff bandwidth and reaching marginalized community members who have limited interactions with healthcare systems in similar low-income rural settings. We also identified the mayor and the lieutenant governor as community partners who helped incentivize case reporting, manage infected pigs and meat, and support the development of local ordinances related to animal husbandry and sanitation. In the RT rollout, we also found municipal environmental health workers essential for addressing gaps in public animal management services by disseminating public health information, receiving reports, providing incentives, and serving as representatives of the environmental perspective in One Health programs. Our findings emphasize the need to study the external implementation context to identify key players, especially those outside health systems, in community-engaged surveillance systems, as well as to conduct continuous evaluation to identify emerging leaders to address barriers.

This study faced limitations exacerbated by the COVID-19 pandemic and political turmoil, resulting in significant delays and high turnover among health care workers, and compromising our ability to complete all planned measures in phase 3 RT rollout. Also, lightly-infected pigs may have been missed during tongue or meat examination, as the sensitivity-probability of truly identifying infected pigs increases depending on cyst density, ^26, 58, 59^ which may have resulted in underreporting. Routine and established meat inspection systems have been reported to be more sensitive than lingual examination; however, sensitivity is also affected by infection intensity, the number of incisions, and the inspector’s skill.^27, 60^ Social factors such as community norms and ‘street-level diplomacy’ influence hesitancy to report as well as compliance with safety rules.^61^ Given these limitations, we recommend future RT implementation studies include strategies across each of the sectors involved in RT delivery, including human (e.g., health workers, preventing open defecation), environmental (e.g., improving water and sanitation), and animal (e.g., meat inspection systems, safe husbandry and animal hygiene).^27^

## CONCLUSION

In this paper, we develop an implementation approach for Ring Treatment (RT) and present results from its multiphase implementation in northern Peru. We found encouraging implementation outcomes, indicating that RT can be a feasible community-engaged intervention to control *T. solium* taeniasis/cysticercosis in low-resource settings. The strategies developed for RT implementation could be applied to community-engaged control interventions for other zoonoses. The study’s formative, pilot, and rollout phases collectively highlight the importance of cross-sectoral collaboration, CHW engagement, adaptive protocol development, and sustained governmental support for successful intervention delivery. Although challenges such as the COVID-19 pandemic, political instability, and resource constraints affected evaluation and maintenance, the implementation of RT in the public health system achieved high community participation, increased awareness, and regional policy changes that support zoonotic disease control. These findings underscore the need for continued investment in One Health approaches—addressing human, animal, and environmental factors—to sustain progress and guide future implementation research in low-income endemic regions.

## Supplementary Material

This is a list of supplementary files associated with this preprint. Click to download.

- RingsImplMainStudyEvalAdditionalDoc1.docx
- RingsImplMainStudyEvalAdditionalDoc1.docx
- RingsImplMainStudyEvalAdditionalDoc3.docx
- RingsImplMainStudyEvalAdditionalDoc3.docx
- RingsImplMainStudyEvalAdditionalDoc2.docx
- RingsImplMainStudyEvalAdditionalDoc2.docx

## Figures and Tables

**Table 1:** Summary of the methods applied in the formative evaluation of RT implementation

Method	Objective	Data collection
Template analysis using the Expanded CFIR for LMIC^35^	Identify barriers and facilitators to RT adoption	Semi-structured interviews with health care leaders (*n* = 169)
Social Network Analysis	Identify key players who will adopt and implement the intervention.	Network surveys with community agents (n = 150), healthcare workers (n = 169), and reps. of external organizations (n = 22)
Process Mapping	Understand the organizational context to inform RT delivery protocols	2 workshops with small district healthcare workers (*n = 15*) and community agents (*n = 11*)

**Table 2:** Re-AIM operationalization across phases 1, 2, and 3 of the multiphase RT implementation.

Set of Strategies	Phase	Mechanisms	Proximal Outcome	Implementation Outcomes		Data collection tools
Identify early adopters	P1	Motivation	Increased human and animal systems rate	Reach	% health care centers participation in training	
Cultivate relationships with animal, human, and environmental stakeholders	P1, P2, P3	Trust building			% staff & CHW attendance at the workshop	Registries
Obtain formal commitments	P1	Authoritative	Increased training attendance rate			
Conduct participatory educational meetings and community outreach	P2, P3	Motivation	Increased workshop attendance		# community members attendance	
Involve and train community health workers and community leaders in implementation efforts	P2, P3		Increased community participation		% community participation in treatment	
Alter the community incentive structure	P2, P3	Motivation			# reports of infected animal/meat	
Alter surveillance system’s structure	P2, P3		Increased reporting rate			
Assess readiness and identify barriers and facilitators	P1	Adoption	Increased response from public health, community, and animal systems	Effectiveness	Seroprevalence of pig cysticercosis	Pig blood drawn and assessment of antibodies against cysticercosis
Stage implementation scale-up	P1, P2, P3	Continuous improvement	Increased RT implementation adoption	Adoption	# health facilities with established procedures for RT	Registries and surveys
Provide consultation for development of regulatory documents & local ordinances	P2, P3	Authoritative	Increased acceptance of RT implementation			
Provide ongoing consultation to health systems	P2, P3	Guidance	Increased adherence to RT protocol	Implementation	Fidelity: Adherence to the established protocol	Checklist
Capture local knowledge	P2, P3	Adaptation	Increase response time and completion		Time to complete the report response	Checklist
Top-down approach	P2, P3	Accountability	Increased sustainment	Maintenance	# health facilities with established procedures for RT at the end of the project	Registries
		Access to medication	Increased treatment completion			
		Trust building	Increased policy adoption			
		Authoritative	Increased funding provision			
Promote network weaving	P2, P3	Relationships	Increased multisectoral collaboration			

Multiple methods, including qualitative and quantitative methods, are explained below.

**Table 3:** Summary of the population treated among the five Ring Treatments

# of Rings	Target population	Treated population	%
1	70	46	66%
2	156	92	59%
3	92	79	86%
4	41	32	78%
5	224	191	85%
	**583**	**440**	**75%**

**Table 4:** RT implementation evaluation outcomes with the RE-AIM framework

Construct	Results	Source
Reach	100% (n = 7/7) of health facilities participating in training and RT delivery	Registries
	52.7% (n = 58/110) of facility staff participating in training and RT delivery	
	242 participants who attended the evidence workshop	
	1 report of infected pork or pig was received 1 report was positively confirmed and received an incentive	
	86.9% (n = 60/69) % of eligible people received NSM treatment	
Adoption	100% (n = 7/7) of health facilities with established procedures for RT	Checklists
Implementation		
	Facilitators: strong collaboration among health personnel, CHWs, and community members (a local butcher, the governor, and animal health coordinators). Barriers: insufficient dissemination, inadequate training, logistical challenges, and personnel shortages.	Interviews
Maintenance	100% (n = 7/7) of health facilities have established procedures for RT.	Checklists

## Data Availability

The datasets generated and analyzed in the current study are not publicly available due to restrictions imposed by the Human Research Ethics Committee. Requests to view these data can be made to the corresponding author and the ethics committee on reasonable request.

## ABBREVIATONS

CHWs: Community Health Workers
CC: Cysticercosis
CFIR: Consolidated Framework for Implementation Research
LMIC: Low and Middle-Income Countries
NCC: Neurocysticercosis
MDA: Mass-drug administration
NSM: Niclosamide
RE-AIM: Reach, Effectiveness, Adoption, Implementation, Maintenance Framework
RT: Ring Treatment
*T.Solium*: *Taenia Solium*
